# Effects of Lipid-Modifying and Other Drugs on Lipoprotein(a) Levels—Potent Clinical Implications

**DOI:** 10.3390/ph16050750

**Published:** 2023-05-16

**Authors:** Amalia Despoina Koutsogianni, George Liamis, Evangelos Liberopoulos, Petros Spyridonas Adamidis, Matilda Florentin

**Affiliations:** 1 Department of Internal Medicine, Faculty of Medicine, University of Ioannina, 45110 Ioannina, Greece; amaliadespoina.koutsogianni@gmail.com (A.D.K.); gliamis1@gmail.com (G.L.); padam7@yahoo.com (P.S.A.); 2 1st Propaideutic Department of Medicine, School of Medicine, National and Kapodistrian University of Athens, Laiko General Hospital, 11527 Athens, Greece; vaglimp@yahoo.com

**Keywords:** antisense oligonucleotides, lipoprotein(a), PCSK9 inhibitors, small interfering RNAs, statins

## Abstract

The past few years have shown an ongoing interest in lipoprotein(a) (Lp(a)), a lipid molecule that has been proven to have atherogenic, thrombogenic, and inflammatory properties. Several lines of evidence, indeed, have demonstrated an increased risk of cardiovascular disease as well as calcific aortic valve stenosis in patients with elevated Lp(a) levels. Statins, the mainstay of lipid-lowering therapy, slightly increase Lp(a) levels, while most other lipid-modifying agents do not significantly alter Lp(a) concentrations, except for proprotein convertase subtilisin/kexin type 9 (PCSK9) inhibitors. The latter have been shown to reduce Lp(a) levels; however, the clinical significance of this effect has not been clearly elucidated. Of note, the pharmaceutical lowering of Lp(a) may be achieved with novel treatments specifically designed for this purpose (i.e., antisense oligonucleotides (ASOs) and small interfering RNAs (siRNAs)). Large clinical trials with cardiovascular outcomes with these agents are ongoing, and their results are eagerly awaited. Furthermore, several non-lipid-modifying drugs of various classes may influence Lp(a) concentrations. We have searched MEDLINE, EMBASE, and CENTRAL databases up to 28 January 2023 and summarized the effects of established and emerging lipid-modifying drugs and other medications on Lp(a) levels. We also discuss the potent clinical implications of these alterations.

## 1. Introduction

Lipoprotein(a) (Lp(a)) is a plasma lipoprotein that consists of a low-density lipoprotein (LDL)-like particle in which apolipoprotein B100 (apoB100) is linked by a single disulfide bond to a unique plasminogen-like glycoprotein, i.e., apolipoprotein(a) (apo(a)) [1]. Lp(a) not only promotes atherosclerosis but also has inflammatory, oxidative, thrombotic, and antifibrinolytic properties [1]. 

The concentration of Lp(a) is almost exclusively genetically determined with an autosomal codominant inheritance pattern and strongly determined by a single gene, the *LPA* gene [1]. Serum Lp(a) levels are inherited in an autosomal codominant manner [1]. Greater than 90% of the variability in Lp(a) levels is determined by a single gene, i.e., the *LPA* gene [1]. By the age of 2, the *LPA* gene is fully expressed [2]. Adult Lp(a) levels are usually reached by approximately the age of five but may further increase until adulthood [2]. Elevations in Lp(a) levels, though, may be observed in pregnancy and menopause [3,4]. Ethnicity also affects Lp(a) concentrations, with median Lp(a) increasing sequentially in Chinese, White, South Asian, and Black individuals [5]. Furthermore, sex influences Lp(a) concentrations; specifically, women have ∼5–10% higher levels compared with men in both Blacks and Whites [6]. Overall, Lp(a) levels are stable over time and are not generally affected by diet, physical activity, or other environmental factors [7].

**Figure 1:**
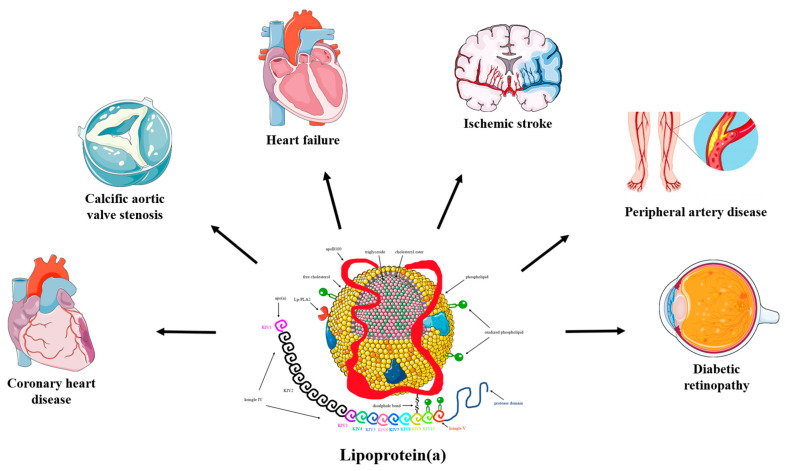
The pathogenicity of lipoprotein(a). Convincing evidence has emerged from pathophysiological, epidemiological, and genetic studies on the causality of high serum lipoprotein(a) (Lp(a)) levels as a potent risk factor for coronary heart disease (CHD), ischemic stroke, peripheral artery disease, heart failure, calcific aortic valve stenosis (CAVS), mitral valve stenosis, and retinopathy in patients with diabetes. Mendelian randomization and genome-wide association studies support the role of Lp(a) as an independent cardiovascular risk factor.

Lp(a) is a well-recognized, independent risk factor for atherosclerotic cardiovascular disease (ASCVD) [8,9]. Evidence from experimental, observational, and genetic studies has demonstrated that increased Lp(a) is associated with increased risk for coronary heart disease (CHD), ischemic stroke, peripheral artery disease, heart failure, calcific aortic valve stenosis (CAVS), mitral valve stenosis, and possibly retinopathy in diabetic patients [1,10,11,12,13,14,15,16,17,18] (Figure 1). On the contrary, elevated Lp(a) concentrations do not appear to be a risk factor for venous thromboembolism [7,8], as previously considered, whereas very low Lp(a) levels have been linked to an increased risk of incident type 2 diabetes mellitus (T2DM) [19]. 

The thresholds commonly used for high Lp(a) are >30 mg/dL or >50 mg/dL (>75 nmol/L or >125 nmol/L, respectively), as levels above these thresholds have been associated with increased ASCVD risk. Importantly, about 20% of the general population have Lp(a) > 50 mg/dL (>125 nmol/L) [7]. Recent guidelines recommend that Lp(a) should be measured in every adult at least once in a lifetime to identify those with very high Lp(a) levels, i.e., >180 mg/dL (>450 nmol/L), and these individuals are considered to have similar ASCVD risk as individuals with heterozygous familial hypercholesterolemia (FH) [20,21,22]. In addition, Lp(a) measurements should be considered in patients with a personal and/or family history of premature ASCVD or high Lp(a) and also for cardiovascular risk reclassification in those at moderate and high ASCVD risk [20,22]. Screening is also recommended for young adults with ischemic stroke and no other identifiable risk factors [20,22].

Currently, there are no medications specifically approved for Lp(a)-lowering [23,24,25]. However, clinical trials in the advanced stages of development with novel agents targeting Lp(a) are ongoing. Interestingly, some therapeutic agents of various drug classes appear to affect Lp(a) concentrations [23,24,25]. In this narrative review, we discuss the effects of current and future lipid-modifying and non-lipid-modifying medical interventions on Lp(a) levels and their potent clinical implications.

## 2. Methods

We searched MEDLINE, EMBASE, and CENTRAL databases up to 28 January 2023 using the following terms: lipoprotein(a), cardiovascular risk, hypolipidemic treatment, statins, ezetimibe, proprotein convertase subtilisin/kexin type 9 inhibitors, lipoprotein apheresis, antisense oligonucleotides (ASOs), small interfering RNAs, tamoxifen, thyroid, aspirin, sex hormones, anti-inflammatory, tocilizumab, interleukin-6 inhibitors, and diet. Clinical trials, review articles, and case reports were assessed, whereas the references of these articles were scrutinized for other relevant articles.

## 3. Lp(a)-Lowering Therapies

### 3.1. Effects of Lipid-Modifying Interventions on Lp(a) Levels

#### 3.1.1. Lipoprotein Apheresis

The most effective clinically available intervention for Lp(a)-lowering is lipoprotein apheresis (LA) [26]. Lp(a) levels are reduced by 70–80%, acutely, after treatment, but rebound elevations between apheresis sessions, which are typically weekly, biweekly, or less frequently, result in a mean interval Lp(a) reduction of 25–40%, depending on the course and baseline Lp(a) levels [26]. LA is infrequently used worldwide, except in Germany, and long-term studies in patients with high Lp(a) undergoing LA suggest that this therapy may reduce 5-year cardiovascular risk by up to 86% [27]. However, LA remains a semi-invasive, time-consuming, and chronic-expensive therapy with variable adherence [28]. The Hellenic consensus panel on the clinical use of LA proposes that LA treatment still has a role in individuals with hypercholesterolemia, including heterozygous FH for the secondary prevention of ASCVD, if they are under a maximum-tolerated lipid-lowering treatment (LLT) and have Lp(a) levels > 100 mg/dL (>238 nmol/L) [29]. Furthermore, LA may be implemented in individuals with severely elevated Lp(a) (>180 mg/dL (>430 nmol/L)) for the primary prevention of ASCVD if they have LDL cholesterol (LDL-C) > 190 mg/dL under a maximum LLT as well as in individuals with diabetes and Lp(a) > 180 mg/dL (>430 nmol/L) with (a) ASCVD, (b) chronic kidney disease stage 4 or 5, or (c) 24-urine albumin > 300 mg [29]. 

#### 3.1.2. Statins

The effect of statin treatment on Lp(a) levels remains an area of controversy. Clinical trials of statin therapy have shown mixed results regarding their impact on Lp(a) levels. A large meta-analysis of six trials, including 5256 patients randomized to receive various statins or a placebo, indicated that most statins may increase Lp(a) by an average of 8–24%, although significant heterogeneity in the response of Lp(a) levels to statin administration, as well as between different statins, has been reported [30]. However, another meta-analysis of 39 trials, including 24,448 patients randomized to receive various statins or a placebo, indicated a non-significant 0.1% increase in Lp(a) in the statin groups (vs. the placebo), with no significant differences among the statins or different intensities of statins [31]. Nonetheless, statin therapy has demonstrated a clinical benefit in patients with elevated Lp(a) in both primary and secondary preventions [32,33]. A meta-analysis of seven randomized controlled trials, including 29,069 patients with high Lp(a) levels and a history of cardiovascular events, concluded that those with Lp(a) levels > 50 mg/dL, at the baseline or in treatment with statins, are at a significantly higher risk of ASCVD as compared with those with <30 mg/dL, independent of other conventional risk factors [32]. The data from this meta-analysis suggest that patients with raised concentrations of Lp(a) (>50 mg/dL), representing about 25% of those with previous cardiovascular disease, are at substantial residual risk, despite statin treatment [32]. The mechanisms by which statins influence Lp(a) levels remain unclear [30]. Statins elevate the expression of *LPA* mRNAs, as well as the synthesis and secretion of the apo(a) protein in HepG2 cells, which may result in an increase in Lp(a) levels [30]. Moreover, statins activate the expression of PCSK9 genes and increase PCSK9 levels, which then enhance Lp(a) production [34,35,36].

#### 3.1.3. Niacin

Niacin decreases Lp(a) in a dose-dependent manner by approximately 30–40% on average [37] but only by 18% in those with the highest Lp(a) levels [38]. The effect of niacin is likely due to a decreased apo(a) production rate [39]. Importantly, studies with cardiovascular outcomes showed no benefit of adding niacin to statins [40,41]. Moreover, niacin use is limited by side effects, such as flushing, gastrointestinal discomfort, and new-onset diabetes [40,41].

In a study conducted a few years ago, the addition of PCSK9 inhibitors to niacin resulted in a 15% additional reduction in Lp(a) compared with that achieved with niacin monotherapy [42].

#### 3.1.4. Ezetimibe

The effect of ezetimibe on Lp(a) is not clear. In a meta-analysis of seven trials of ezetimibe monotherapy, ezetimibe significantly reduced Lp(a) levels by 7.1% [43]. In contrast, in another meta-analysis, ezetimibe, either as monotherapy vs. placebo or in combination with statin vs. statin alone, did not significantly alter Lp(a) concentrations [43]. The mechanisms by which ezetimibe may influence Lp(a) levels have not been clearly elucidated [43]. Ezetimibe likely has anti-inflammatory activity and may affect Lp(a) production, as Lp(a) is an acute-phase reactant [43]. 

#### 3.1.5. PCSK9 Inhibitors

PCSK9 monoclonal antibodies, i.e., alirocumab and evolocumab, reduce Lp(a) levels by approximately 20–30% [44,45,46]. In the two major PCSK9 inhibitor trials, the FOURIER trial, with evolocumab, and the ODYSSEY OUTCOMES trial, with alirocumab, both PCSK9 inhibitors were associated with an approximately 25% reduction in the risk of major adverse cardiovascular events (MACEs) [47,48]. Secondary analyses of these trials indicated that the main factor determining both the risk for MACEs in the placebo groups as well as the reductions in cardiovascular outcomes in patients treated with PCSK9 inhibitors was Lp(a) concentrations [49]. Indeed, in the ODYSSEY OUTCOMES trial, patients with a recent acute coronary syndrome, LDL-C near 70 mg/dL in their optimized statin treatment, and Lp(a) levels that were at least mildly elevated (≥13.7 mg/dL) derived substantial clinical benefit from the treatment with alirocumab [49]. In contrast, patients with LDL-C near 70 mg/dL and lipoprotein(a) < 13.7 mg/dL had no reduction in MACEs with alirocumab, whereas patients with higher LDL-C levels derived consistent clinical benefit from alirocumab treatment, irrespective of the level of Lp(a) [49]. The exact mechanism by which PCSK9 inhibitors reduce Lp(a) levels remains unclear [50]. Current hypotheses include increased clearance of Lp(a) particles via the LDLR, increased clearance of Lp(a) via other receptors (the LDL receptor-related protein 1, the cluster of differentiation 36 receptor, toll-like receptor 2, scavenger receptor-B1, and plasminogen receptors), as well as a reduction in apo(a) production, secretion, and/or assembly [50].

Inclisiran, a small interfering RNA (siRNA) targeting intracellular PCSK9, has been shown to reduce Lp(a) levels by approximately 20% [51]. The clinical significance of this finding warrants further investigation.

#### 3.1.6. Fibrates

Bezafibrate has been shown to reduce Lp(a) levels by approximately 13–39% [52]. A similarly modest, but not statistically significant, effect was demonstrated for gemfibrozil [53]. Thus, fibrates do not appear to influence Lp(a) concentrations significantly [54]. 

#### 3.1.7. Lomitapide

Lomitapide, a microsomal triglyceride transfer protein inhibitor, reduces Lp(a) levels by 15–19% [55]. The possible mechanism for Lp(a)-lowering is the decrease in very-low-density lipoprotein (VLDL) and chylomicron synthesis via inhibition of MTP [55]. MTP is located in the endoplasmic reticulum of hepatocytes and enterocytes and is most likely responsible for transferring triglycerides to nascent apoB as it enters the lumen of the endoplasmic lumen [55]. Consequently, MTP inhibition seems to control the number of apoB-containing lipoprotein particles secreted into the bloodstream, including Lp(a) particles [55]. However, its use is restricted to patients with homozygous familial hypercholesterolemia [55] and is not indicated for Lp(a)-lowering [56].

#### 3.1.8. Mipomersen

Mipomersen is an ASO-targeting apoB100 [57]. In clinical trials, mipomersen significantly reduced Lp(a) levels by 21–39% [57,58]. The mechanism for Lp(a)-lowering seems to involve a decrease in the availability of apoB100 for Lp(a) assembly [59]. However, mipomersen has been withdrawn from the market due to side effects, which mainly include an increased risk of hepatic steatosis and hepatic enzyme elevation [57,58,59].

#### 3.1.9. Cholesteryl Transfer Protein (CETP) Inhibitors 

CETP mediates the transfer of cholesteryl esters from high-density lipoprotein (HDL) to apoB100-containing particles, including VLDL and LDL, in exchange for triglycerides [60,61,62,63]. Apart from raising HDL cholesterol [60,61,62,63], CETP inhibitors also decrease apoB100, LDL cholesterol, and Lp(a) levels (by approximately 24–36%) [60,61,62,63]. Clinical outcome trials of four CETP inhibitors have been completed, including torcetrapib, dalcetrapib, evacetrapib, and anacetrapib [64]. However, they either had only modest clinical benefits or even clinical futility after prolonged treatment, whereas torcetrapib increased mortality [64]. Thus, CETP inhibitors are not currently approved for clinical use. 

We should note, though, that treatment with 5 or 10 mg of obicetrapib, another CETP inhibitor, robustly reduced LDL-C, apoB, non-HDL-C, and Lp(a) (33.8% vs. 56.5%, respectively) and increased HDL cholesterol and apoA1, as an adjunct to high-intensity statins, compared with the placebo in a randomized, double-blind, placebo-controlled, phase 2 trial with a high safety and tolerability profile [65]. Additional phase 3 investigations, including a cardiovascular outcomes trial, are currently underway to further assess the safety and clinical benefits of obicetrapib [65]. 

#### 3.1.10. Bempedoic Acid

Bempedoic acid inhibits the adenosine triphosphate citrate lyase upstream from the 3-Hydroxy-3-methylglutaryl-coenzyme A (HMG-CoA) reductase and decreases cholesterol biosynthesis in the liver. Subsequently, bempedoic acid upregulates LDL receptors and increases the clearance of LDL particles [66]. In a phase 2 trial, bempedoic acid had no significant effect on Lp(a) levels [67]. Similarly, the combination of bempedoic acid with evolocumab was not superior to evolocumab monotherapy in terms of Lp(a) change from the baseline [68]. In a phase 3, randomized, double-blind, placebo-controlled trial (the CLEAR OUTCOMES trial), bempedoic acid (180 mg daily) was associated with a statistically significant and clinically meaningful reduction in the risk of MACEs in 14,014 patients at high risk for ASCVD with documented statin intolerance and elevated LDL cholesterol levels (≥100 mg/dL) [69].

#### 3.1.11. Bile Acid Sequestrants

Bile acid sequestrants (colesevalem, cholestyramine, and colestipol) interrupt the enterohepatic circulation of bile acids [70]. Thus, they increase the conversion of cholesterol into bile acids and upregulate LDL receptors. These drugs have not been shown to affect Lp(a) levels [70].

#### 3.1.12. ASO and siRNA Agents 

**Figure 2:**
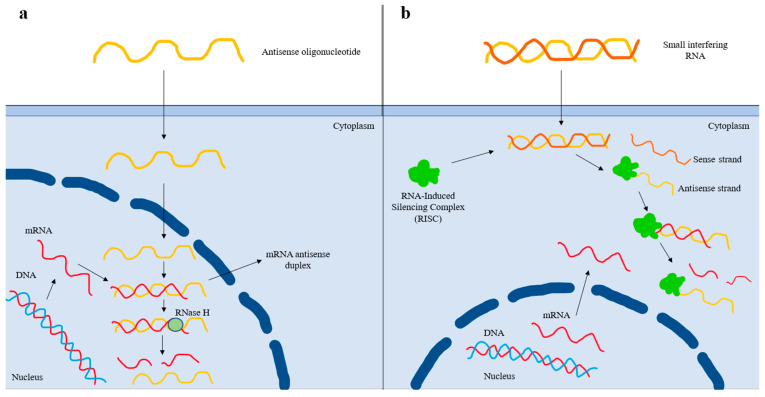
Mechanism of action of antisense oligonucleotide and small interfering RNA-based therapies. (**a**) After entering the nucleus, the antisense oligonucleotide (ASO) binds to the complementary sequence of the targeted mRNA. The resulting mRNA antisense duplex is recognized by RNase H, which cleaves the mRNA and, thus, prevents protein translation. (**b**) After the small interfering RNA (siRNA) enters the cell, it is recognized by the RNA-Induced Silencing Complex (RISC), which removes the sense strand. The resulting complex binds to the complementary mRNA sequence and degrades it, thus preventing protein translation. The antisense strand bound to the RISC forms a recyclable, stable complex, and, as a result, its action against target mRNA strands can be repeated. Thereby, less-frequent dosing of siRNAs is needed compared to that of ASOs.

In the era of RNA-based therapies, ASOs and siRNAs targeting Lp(a) are currently in clinical development [71]. Pelacarsen (formerly IONIS-APO(a)-LRX, AKCEA-APO(a)-LRX, TQJ230) is a second-generation ASO that binds to its target complementary RNA sequence via base pairing, thereby leading to the degradation of the apo(a) mRNA strand and reduced Lp(a) production [72] (Figure 2). Pelacarsen is conjugated to N-acetylgalactosamine (GalNAc), which enables specific targeting to the hepatocytes, thus providing increased drug potency, less systemic toxicity, and less-frequent dosing [72]. In a phase 2 trial, a randomized, double-blind, placebo-controlled, dose-ranging trial involving 286 patients with established ASCVD and screening Lp(a) levels of at least 60 mg/dL (150 nmol/L), pelacarsen effectively reduced Lp(a) levels in a dose-dependent way (i.e., 35% at the dose of 20 mg every 4 weeks, 56% at 40 mg every 4 weeks, 58% at 20 mg every 2 weeks, 72% at 60 mg every 4 weeks, and 80% at 20 mg every week) [73]. The most common adverse effect was injection site reactions [73]. A phase 3 cardiovascular outcome trial (HORIZON trial; ClinicalTrials.gov Identifier: NCT04023552) with pelacarsen is currently ongoing in patients with elevated baseline Lp(a) levels (>70 mg/dL) and established ASCVD (a prior history of myocardial infarction, ischemic stroke, or symptomatic peripheral artery disease). Contrary to phase 2 studies, the dose of 80 mg once monthly subcutaneously is being evaluated.

In contrast to ASOs, siRNAs are double-stranded RNA molecules that dissociate once inside the cell, and the antisense strand is inserted into the RISC (RNA-Induced Silencing Complex) [74]. The antisense strand binds to its homologous target mRNA sequence, leading to its degradation [74]. The antisense strand bound to the RISC forms a recyclable, stable complex, and, as a result, its action against target mRNA strands can be repeated [74]. Thereby less-frequent dosing of siRNAs is needed compared to that of ASOs [74] (Figure 2). Olpasiran (formerly AMG890, ARO-LPA), a GalNAc-conjugated siRNA agent against apo(a), reduced Lp(a) by up to >90% at doses ≥ 9 mg, with its effects persisting for an average of 3 to 6 months with no safety concerns, in a phase 1 clinical trial [75]. Equally encouraging are the results obtained from the phase 2 randomized, double-blind, placebo-controlled, dose-finding trial (a double-blind, randomized, placebo-controlled phase 2 study to evaluate the efficacy, safety, and tolerability of AMG 890 (a GalNAc-conjugated small interfering RNA in subjects with elevated lipoprotein(a); the OCEAN(a)-DOSE trial; NCT04270760) involving patients with established ASCVD and Lp(a) levels of >150 nmol/L. In this trial, 281 patients were randomly assigned to receive one of four doses of olpasiran (10 mg every 12 weeks, 75 mg every 12 weeks, 225 mg every 12 weeks, or 225 mg every 24 weeks) or a matching placebo administered subcutaneously [76]. At 36 weeks, the olpasiran therapy had significantly and substantially reduced the Lp(a) levels in a dose-dependent manner, resulting in placebo-adjusted mean percent changes of −70.5% with the 10 mg dose, −97.4% with the 75 mg dose, −101.1% with the 225 mg dose administered every 12 weeks, and −100.5% with the 225 mg dose administered every 24 weeks [76]. The overall incidence of the adverse events was similar across the trial groups, with the most common olpasiran-related adverse events being injection site reactions, primarily pain [76]. Further research with olpasiran, including cardiovascular outcomes clinical trials, are warranted to assess the role of this agent in clinical practice. Currently, a phase 3 double-blind, randomized, placebo-controlled trial assessing the impact of olpasiran on the risk for CHD death, myocardial infarction, or urgent coronary revascularization in patients with ASCVD and elevated Lp(a) levels (≥200 nmol/L) is ongoing (Olpasiran Trials of Cardiovascular Events and Lipoprotein(a) Reduction (OCEAN(a))—Outcomes Trial; NCT05581303).

Apart from olpasiran, another GalNAc-conjugated siRNA against the apo(a) mRNA, SLN360, reduced circulating Lp(a) by up to 95% in cynomolgus monkeys [77]. A phase 1 trial of SLN360 (NCT04606602) reported a dose-dependent Lp(a) reduction of up to 98% in 32 patients with elevated Lp(a) levels and no known cardiovascular disease [78]. Most patients treated with SLN360 experienced mild to moderate injection site reactions, but further study to determine the safety and efficacy of this siRNA is necessary [78].

Moreover, a phase 1 trial investigating the effect of LY3819469, another GalNAc-conjugated siRNA against the apo(a) mRNA, in patients with elevated Lp(a) levels and increased cardiovascular risk is now ongoing (a study of LY3819469 in healthy participants; NCT04914546).

### 3.2. Effects of Other Drugs on Lp(a) Levels

#### 3.2.1. Sex Hormone Therapies

Estrogen and its analogs reduce the transcription of the *LPA* gene [79]. While endogenous sex hormones do not substantially affect Lp(a) levels, postmenopausal hormone replacement therapy can lower Lp(a) levels by approximately 20–25% [80,81]. A systematic review and meta-analysis of 10 clinical trials, which included 2049 postmenopausal women, suggested that anti-estrogen therapy significantly reduced Lp(a) levels by approximately 5.92% [82]. However, adverse effects (e.g., breast cancer, stroke, and thrombosis) may outweigh potential cardiovascular benefits [83,84]. 

Although previous data suggested that testosterone treatment may lower Lp(a) levels [85], randomized control trials did not confirm this effect [86,87].

Tamoxifen administration in patients with breast cancer has been found to reduce Lp(a) levels by approximately 40% [88,89].

#### 3.2.2. Thyroid Hormone Therapies

Lp(a) levels are decreased in hyperthyroidism and increased in hypothyroidism [90]. Thyromimetic agents (e.g., eprotirome) have been shown to reduce Lp(a) levels [90]. However, in previous programs in animals, these agents were discontinued because of significant adverse effects, such as elevations in liver enzymes and cartilage side effects [90]. Liver-selective thyromimetic agents, such as the thyroid hormone receptor agonist eprotirome and resmetirom (MGL-3196), a liver-directed, orally active thyroid hormone receptor beta agonist, under study for the treatment of nonalcoholic steatohepatitis, reduce Lp(a) levels (by 32.3% and 37.9%, respectively) without extrahepatic adverse events [91,92,93]. In a recent systematic review and meta-analysis, the treatment of overt hyperthyroidism with thyroidectomy, antithyroid drugs, or radioactive iodine increased Lp(a) levels by 20–25%, whereas the treatment of overt and subclinical hypothyroidism with levothyroxine decreased Lp(a) levels by 5–20% [90]. 

#### 3.2.3. Growth Hormone Replacement Therapy

Growth hormone (GH) replacement therapy has been previously found to increase Lp(a) levels by 25–100% [94]. The increase in the Lp(a) levels induced by GHs may partly contribute to the increased ASCVD risk in patients with acromegaly [94]. In a recent prospective observational study, GH replacement therapy in men with GH deficiency resulted in a significant increase in Lp(a) levels (mean: from 27.4 nmol/L to 34.3 nmol/L) [95]. However, long-term studies with GH replacement therapy in GH deficiency are warranted to address these questions.

#### 3.2.4. Aspirin

The available data regarding the effects of aspirin on Lp(a) concentrations and any potent clinical implications of these alterations are scarce. In a post hoc analysis of the Women’s Health Study, a randomized primary prevention trial, 25,131 healthy White women with Lp(a) > 65 mg/dL received aspirin (100 mg every other day) or a placebo [96]. The treatment with aspirin did not significantly reduce cardiovascular events over a 10-year period (the age-adjusted hazard ratio was 0.75 (the 95% CI: 0.48–1.18, *p* = 0.22)) [96]. In a subgroup analysis, though, carriers of the rs3798220 variant, which is associated with high circulating Lp(a) levels, with a baseline median Lp(a) ≈ 80 mg/dL, significantly benefitted from aspirin treatment (HR 0.44 and the 95% CI: 0.20–0.94, *p* = 0.03) [96]. This may imply that the benefit of aspirin treatment depends on the Lp(a) levels, but this warrants validation in further studies [96]. Thus, individuals with high Lp(a) levels might be considered for aspirin therapy if they also have other indications for aspirin therapy (e.g., very high ASCVD risk and low bleeding risk) [7,97]. 

#### 3.2.5. Anti-Inflammatory Agents

Tocilizumab, a monoclonal antibody targeting interleukin-6, reduced Lp(a) levels by approximately 30–40% when administered in rheumatoid arthritis patients [98]. The *LPA* gene promoter contains functional interleukin 6-responsive elements; thus, this effect of tocilizumab may be attributed to its inhibitory action in this region of the gene [99]. 

On the contrary, protease inhibitors and antiretroviral therapy have been associated with increased Lp(a) levels when administered in human immunodeficiency patients with baseline Lp(a) levels > 20–30 mg/dL [100].

### 3.3. Effects of Dietary Intervention and Physical Activity on Lp(a) Levels

Lifestyle changes, including low-fat diets, have no significant effect on Lp(a) levels [101,102]. Some, but not all, studies reported that isocaloric replacement of dietary saturated fats with carbohydrates or unsaturated fats increased Lp(a) levels by approximately 8–20% [102,103,104]. On the contrary, low-carbohydrate, high-saturated fat diets and diets enriched with walnuts or pecans decreased Lp(a) levels by 15% and 6–15%, respectively [102,105]. Other studies have investigated the effect of dietary supplements (L-carnitine and coenzyme Q10) and specific foods (coffee, tea, and alcoholic beverages, especially red wine) on Lp(a) levels and have shown modest decreases of 10–30% with these interventions [101,106]. Finally, vitamin C, in supplementary (1 g/day) or pharmacologic doses (4.5 g/day), has a neutral effect on plasma Lp(a) levels, as shown in a recent meta-analyses [107,108].

Most studies suggest that physical activity has no or minimal impact on Lp(a) levels, although there are conflicting data, particularly among younger or diabetic populations in whom physical fitness correlated inversely with Lp(a) levels and decreased CVD risk [109,110].

### 3.4. Effects of Bariatric Surgery on Lp(a) Levels

A prospective observational study of 59 patients with severe obesity (with a median Body Mass Index (BMI) of 48 kg/m^2^) undergoing metabolic surgery (thirty-one had Roux-en-Y gastric bypass, nineteen had sleeve gastrectomy, and nine had omega loop bypass) demonstrated a significant increase in Lp(a) levels at 12 months following bariatric surgery (10.2 (3.8–31.9) vs. 16.9 (4.9–38.6) mg/dL, *p* = 0.002) [111]. These results may suggest an increased synthetic liver capacity, leading to improved production and secretion of Lp(a) and, thus, to higher plasma levels [111].

The effects of all the aforementioned agents on Lp(a) concentrations and their possible mechanisms of action are summarized in Table 1 and Table 2, respectively. 

Several lines of evidence suggest that large absolute reductions in Lp(a) concentrations of ~100 mg/dL are needed to achieve meaningful reductions in ASCVD risk [7,112,113]. Therefore, only those drugs that are specifically designed for the purpose of Lp(a)-lowering could be expected to have beneficial effects on ASCVD outcomes. Ongoing and future studies addressing this question are pending.

## 4. Conclusions

Lp(a) is a novel risk factor for ASCVD and CAVS, and this risk increases linearly with Lp(a) concentrations. It appears that robust reductions in Lp(a) concentrations should be achieved for meaningful reductions in cardiovascular outcomes. In patients with high Lp(a) levels, more aggressive LDL-C targets, compared with those recommended for individuals with otherwise the same ASCVD risk, are considered. Thus, our initial approach to reduce ASCVD risk in patients with elevated Lp(a) is to further reduce LDL-C. However, several promising agents are in the late stages of development, and the results of phase 3 outcome studies are eagerly awaited. Several other agents, either lipid-modifying or of other drug classes, also alter Lp(a) levels. However, there is no solid evidence that these changes have clinical significance, with the possible exception of PCSK9 inhibitors.

## Figures and Tables

**Table 1:** Current and emerging lipoprotein(a)-lowering therapies.

Lipoprotein(a)-Lowering Therapy	Lipoprotein(a) Effect
Lipoprotein apheresis	Acute decrement of 70% to 80%. Regular apheresis can translate into a mean Lp(a) reduction between 25% and 40% [26].
Statins	Mixed results from clinical trials. Statins may increase Lp(a) by an average of 8% to 24% [30,31].
Niacin	Potential Lp(a)-lowering effect by 30% to 40% [37]
Ezetimibe	Neutral effect up to a modest 7% reduction in Lp(a) levels [43]
Proprotein convertase subtilisin/kexin type 9 (PCSK9) inhibitors: Monoclonal antibodies (evolocumab, alirocumab) Small interfering RNA (inclisiran)	Mean reduction of Lp(a) levels between 20% and 30% [45,46,47] Lp(a) reduction by approximately 20% [51]
Fibrates	Potent reduction between 13% and 39% [52]
Lomitapide	Reduction 15% to 19% [55]
Mipomersen	Reduction 21% to 39% [57,58]
Cholesteryl ester transfer protein inhibitors	Reduction 24% to 36% [60,61,62]
Bempedoic acid	Neutral effect [67,68,69]
Bile acid sequestrants	Neutral effect [70]
Antisense oligonucleotides: Pelacarsen	Lp(a) reduction up to 80% [73]
Small interfering RNAs: Olpasiran SLN360	Maximum reduction of >90% [75] Dose-dependent Lp(a) reduction up to 98% [78]
Sex hormone therapies: Estrogen and its analogs Testosterone or anabolic steroids Tamoxifen	Reduction by 20% to 25% [80,81] Neutral effect [86,87] Reduction by approximately 40% [88,89]
Thyroid hormone therapy Eprotirome Resmetirom (MGL-3196)	Reduction up to 32.3% (not approved for clinical use) [91,92,93] Reduction up to 37.9% (not approved for clinical use) [91,92,93]
Growth hormone	Increase by 25% to 100% [94]
Antibodies against interleukin-6 (e.g., tocilizumab)	Reduction between 30% and 40% [98]
Protease inhibitors or antiretroviral therapy	Increase [100]
Low-saturated fat diets	Potential increase between 8% and 20% [102,103,104]
Low-carbohydrate, high-saturated fat diets and diets enriched with walnuts or pecans	Decrement by 15% and 6% to 15%, respectively [102,105]
Dietary supplements (L-carnitine, and coenzyme Q10)	Modest reduction between 10% and 30% [101,106]
Specific foods (coffee, tea, and alcoholic beverages, especially red wine)	Modest reduction between 10% and 30% [101,106]
Vitamin C	Neutral effect on plasma Lp(a) levels [107,108]
Bariatric surgery	Significant increase in Lp(a) plasma levels [111]

**Table 2:** Potential mechanisms of action of current and emerging lipoprotein(a) (Lp(a))-lowering therapies.

Lipoprotein(a) (Lp(a))-Lowering Therapy	Mechanism of Action
Statins	Elevate the expression of LPA mRNA [30]. Elevate the synthesis and secretion of apo(a) protein in HepG2 cells [30]. Activate the expression of PCSK9 genes and increase PCSK9 levels [34,35,36].
Niacin	Decreases apo(a) production rate [39].
Ezetimibe	May influence Lp(a) production via anti-inflammatory activity [43].
PCSK9 inhibitors	Increase the clearance of Lp(a) particles via the LDLR [50]. Increase clearance of Lp(a) via other receptors (LDL receptor-related protein 1, cluster of differentiation 36 receptor, toll-like receptor 2, scavenger receptor-B1, and plasminogen receptors) [50]. Reduce apo(a) production, secretion, and/or assembly [50]
Lomitapide	Decreases the number of apoB-containing lipoprotein particles secreted into the bloodstream, including Lp(a) particles [55].
Mipomersen	Decreases the availability of apoB100 for Lp(a) assembly [59].
CETP inhibitors	Mediate the transfer of cholesteryl esters from high-density lipoproteins to apoB100-containing particles, including VLDL and LDL, in exchange for triglycerides [60,61,62,63]
Bempedoic acid	Upregulates LDLRs and increases the clearance of LDL particles [66].
Antisense oligonucleotides	Bind to its target complementary RNA sequence via base pairing, thereby leading to degradation of the apo(a) mRNA strand and reduced Lp(a) production [72].
Small interfering RNAs	Double-stranded RNA molecules that dissociate once inside the cell, and the antisense strand is inserted into the RISC (RNA-Induced Silencing Complex). The antisense strand binds to its homologous target mRNA sequence, leading to its degradation. The antisense strand bound to the RISC forms a recyclable, stable complex, and, as a result, its action against target mRNA strands can be repeated [74].

Abbreviations: Lp(a), lipoprotein(a); PCSK9, proprotein convertase subtilisin/kexin type 9; apo(a), apolipoprotein(a); LDLR, low-density lipoprotein receptor; apoB, apolipoprotein B; apoB100, apolipoprotein B100; VLDL, very low-density lipoprotein; LDL, low-density lipoprotein; RISC, RNA-Induced Silencing Complex.

## Data Availability

No new data were created or analyzed in this study. Data sharing is not applicable to this article.
